# miR-199a Is Upregulated in GDM Targeting the MeCP2-Trpc3 Pathway

**DOI:** 10.3389/fendo.2022.917386

**Published:** 2022-07-14

**Authors:** Chun-Yi Guan, Jing-Li Cao, Lu Zhang, Xue-Qin Wang, Xu Ma, Hong-Fei Xia

**Affiliations:** ^1^ Reproductive and Genetic Center of National Research Institute for Family Planning, Beijing, China; ^2^ Graduate School, Peking Union Medical College, Beijing, China

**Keywords:** MiR-199a, gestational diabetes mellitus, MeCP2, TRPC3, placenta

## Abstract

Gestational diabetes mellitus (GDM), the most common medical pregnancy complication, has become a growing problem. More and more studies have shown that microRNAs are closely related to metabolic processes. The purpose of this paper is to investigate the role of up-regulation of miR-199a-5p expression in GDM. We found that miR-199a-5p was significantly up-regulated in the placenta of GDM patients compared with normal pregnant women, and expressed in placental villi. miR-199a-5p can regulate the glucose pathway by inhibiting the expression of methyl CpG-binding protein 2 (MeCP2) and down-regulating canonical transient receptor potential 3 (Trpc3). This suggests that miR-199a-5p may regulate the glucose pathway by regulating methylation levels, leading to the occurrence of GDM.

## Introduction

Gestational diabetes mellitus (GDM) is defined as any degree of glucose intolerance onset or first recognized during pregnancy, a disease that develops during the second and third trimesters, and is characterized by marked insulin resistance secondary to placental hormone release (1, 2). The incidence of GDM in China is 11.91%, and the incidence is on the rise (3). Women with GDM are at increased risk of developing gestational hypertension and preeclampsia, and are more likely to experience GDM recurrence in future pregnancies (4–6). GDM is associated with an increased risk of type 2 diabetes (T2D) and cardiovascular disease in mothers and offspring.

MicroRNAs are endogenous non-coding RNA molecules with a length of 19-22 nt (7). Many studies have shown that miRNAs are involved in the pathogenesis of GDM (8–10). miR-199a is differentially expressed specifically in patients with type 1 diabetes, type 2 diabetes and GDM (11, 12). Esteves et al. found that miR-199a is involved in glucose regulation in rat skeletal muscle (13). miR-199a-3p is downregulated in type 2 diabetes and may contribute to diabetic macular edema (14). These studies suggest that miR-199a is involved in blood glucose regulation, but its role in GDM remains unclear.

Methyl-CpG binding protein 2 is a member of the methyl-CpG binding domain (MBD) protein family involved in DNA modification (15). Studies have shown that changes in DNA methylation affect gene expression pathways related to the pathophysiological process of GDM (16). In this study, we report the relationship between miR-199a and GDM and investigate the possible functional role of miR-199a in GDM placenta.

## Materials and Methods

### Sample Collection and Tissue Preparation

137 GDM patients and 158 healthy pregnant women were selected from Beijing Haidian District Maternal and Child Health Hospital. The population information is shown in **Table 1**. There was no significant difference in the age of the GDM compared with the control group, the BMI in the GDM group was significantly higher than that in the control group (*P*<0.05), and the OGTT-2h in the GDM group was significantly higher than that in the control group (*P*<0.05). The selected pregnant women underwent glucose tolerance (OGTT) test according to the IADPSG2010 diagnostic method and standard at 24-28 weeks of gestation. Small pieces of tissue were cut from the placenta from the maternal leaflet and fixed in 10% formaldehyde. GDM placental tissues were divided into four groups according to age: less than 25 years old (Y<25), 25-30 years old (Y25-30), 31-35 years old (Y31-35) and over 35 years old (Y>35). Among the Y<25, there were 23 people in the control group and 10 people in the GDM group; among the Y25-30, there were 72 people in the control group and 58 people in the GDM group; among the Y31-35, there were 43 people in the control group and 40 people in the GDM group; among the Y>35, 20 in the control group, 29 in the GDM group. The collected placental tissue was embedded in paraffin to make a tissue chip, and this tissue chip was used for both immunohistochemistry and *in situ* hybridization experiments. At least 10 samples per group were used for post-staining statistical analysis. 10 samples were taken from each group for Real-time PCR detection. The study complied with the principles stipulated in the Declaration of Helsinki of the World Medical Association to collect clinical specimens, and was approved by the Ethics Committee of the Scientific Research Institute of the National Population and Family Planning Commission (2011–08).

**Table 1:** Basic characteristics of study population according to GDM.

	Control	GDM
Age(years)	29.68±3.6	30.93±3.4
BMI(kg/m2)	21.06±2.9	22.97±3.1*
OGTT-2h	6.26±0.9	9.07±1.3*

*P<0.05.

### *In Situ* Hybridization and Immunohistochemistry

Tissue microarray sections were denatured with 100 μg/mL proteinase K (TransNGS, China) and labeled with digoxigenin (DIG) LNA-miR-199a probe at 40°C overnight. The (AP)-labeled anti-DIG antibody (Roche, Germany) was then incubated in alkaline phosphatase-containing blocking reagent overnight at 4°C. Staining was performed using BCIP/NBT chromogenic substrate (Promega, USA). Three fields of view were randomly selected for each slice, and three different slices were selected for the same sample.

Cells and tissues were incubated with anti-MECP2 polyclonal antibody (GeneTex, USA, 1:200) or anti-5 methylcytosine monoclonal antibody (Santa Cruz, USA, 1:200) overnight at 4°C, then incubated with horseradish peroxidase (HRP)-conjugated goat anti-rabbit IgG or goat anti-mouse IgG (1:500, BiodragonTechnology Co., China) for 1 h. All 137 GDM patients and 158 healthy pregnant women placental tissues were embedded in paraffin to make tissue chips. All samples were used for immunohistochemistry and *in situ* hybridization experiments. The sample selection is Random. The results were quantitatively analyzed by ImageJ software. For cell immunohistochemistry, there were at least three samples in each group, and three viewing angles were selected for each sample to take pictures to count the results.

### Plasmid Construction

A 367 bp MeCP2-3′-UTR containing the miR-199a binding site was ligated to the pGL3 vector. The restriction sites were specific to Xbal and SpeI. (MeCP2-3′UTR-Forward, 5′- GACTAGTTCTCTCTGCTCTGACGGGATTTGT -3′, MeCP2- 3′UTR-Reverse, 5′- GCTCTAGATTCAGAAGCCATGTCCTCAGGT -3′).

The mutant primers were designed using the Easy Mutagenesis System kit (TransGen Biotech, China). The primer sequences are as follows: (MeCP2-3′UTR-mut- Forward, 5′- TATTAGAGGGGAAAAGCTGATTATTGAAGTCAGTTCTCAACAAT -3′, MeCP2-3′UTR-mut-Reverse, 5′- ATAATCAGCTTTTCCCCTCTAATATGTAATTTTAGA -3′). The PMSCV-puro- MeCP2 expression vector was constructed and the MeCP2 CDS sequence was amplified by PCR. The restriction sites were specific to XhoI and EcoRI. (PMSCVpuro- MeCP2-Forward, 5′- CGCGGATCGCCACCATGGTAGCTGGGATGTTAGGGCT -3′, PMSCVpuro- MeCP2- Reverse, 5′- CCGGAATTCTCAGCTAACTCTCTCGGTCACGG - 3′).

### Quantitative Reverse-Transcriptase Polymerase Chain Reaction

Total RNA from tissues and cells was extracted with TRIzol (Invitrogen, USA) and reverse transcribed into cDNA using a reverse transcription kit (Takara Bio, China). miR-199a and U6 probe (Applied Biosystems, USA) were used for reverse transcription and PCR for quantitative miRNA. Each sample was replicated three times, with a minimum of three samples per group. The primer sequences are as follows: MeCP2 - Forward, 5′- TGCAAAGAGGAGAAGATGCCCAGA-3′, MeCP2-Reverse, 5′- GCCTTGGCATGGAGGATGAAACAA -3′, GAPDH-Forward, 5′ - TGGTATCGTGGAAGGACTCA -3′, GAPDH-Reverse, 5′- GTAGAGGCAGGGATGATGTTC-3′.

### Cells Culture and Transfection

JEG-3 was purchased from China National Biomedical Experimental Cell Resource Bank in DMEM complete medium (10% fetal bovine serum, 1% 100 IU/mL penicillin and 100 IU/mL streptomycin), placed in an incubator at 37°C with 5% CO_2_. MiR-199a mimic, miRNA mimic negative control, miR-199a inhibitor or miRNA inhibitor negative control (Gene Pharma, China) JEG-3 cells were transfected according to the instructions of Lipofectamine 2000 Reagent (Invitrogen, USA). The miRNA mimic and inhibitor concentrations were 50 nM, and the plasmid concentration was 0.5 ng/μL.

### Dual-Luciferase Activity Assay

Add 20 μL of cells to the tube, add 100 μL of firefly luciferase (Promega, USA) detection reagent, mix well and use the measured value and record. Then 100 μL of renilla luciferase detection working solution (Promega, USA) was added, and the value was detected and recorded. Relative luciferase activity (Firefly LUC/Renilla LUC) was obtained and the experiment was repeated three times.

### Western Blot Analysis

The tissue and cell proteins were extracted with RIPA Lysis Buffer, the protein concentration was detected with BCA kit (TransGen, China), and 50 μg of protein was electrophoresed on polyacrylamide gel. After electrophoresis, the protein was transferred to PVDF membrane (Amersham, UK), anti-MECP2 polyclonal antibody (GeneTex, USA, 1:500), anti- TRPC3 and anti-SFRP4 polyclonal antibody (1:500, SantCruz Biotechnology Inc., USA), anti-β-ACTIN monoclonal antibody (Abcam, USA, 1:2000) was incubated overnight at 4°C. HRP-conjugated goat IgG was incubated for 1 h. Chemiluminescence reaction was visualized on ECL kit (Millipore, USA). The results were quantitatively analyzed by ImageJ software, with at least 3 samples per group.

### Statistical Analysis

The unpaired Student’s t-test was used to determine the significance between two groups. One-way analysis of variance was used to determine significant differences among the groups. Prism6.0 software was used for illustrations.

## Results

### miR-199a Was Upregulated in the Placenta of GDM Patients

**Figure 1:**
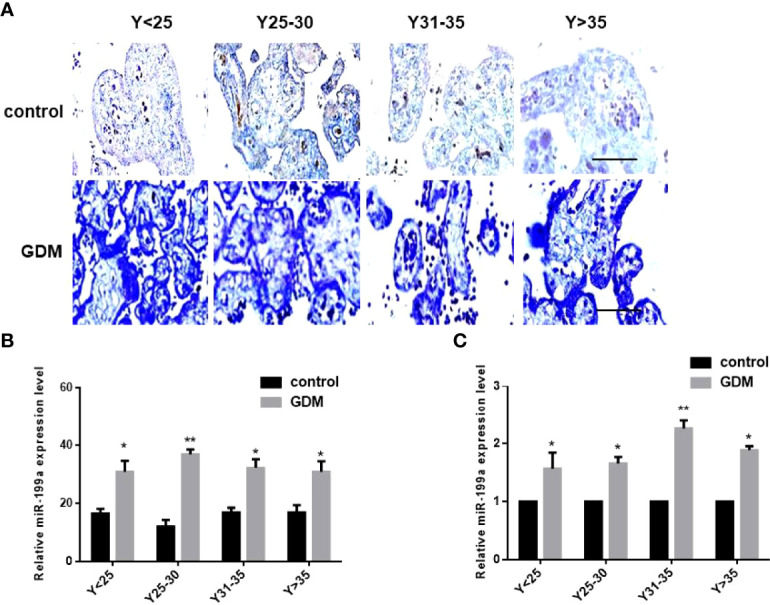
The expression of miR-199a is up-regulated in the placenta of GDM patients. **(A, B)** *In situ* hybridization of miR-199a-specific DIG-labeled LNA probes to detect the localization and expression of miR-199a in the placenta. **(C)** Real-time PCR detection of miR-199a expression in placenta. n=10. The scale bar indicates a distance of 1000μm. *P<0.05; **P<0.01.

In order to exclude the influence of age, we divided the samples into four groups according to the maternal age, 25 years old (Y<25), 25-30 years old (Y25-30), 31-35 years old (Y31-35), and over 35 years old (Y>35). *In situ* hybridization results showed that miR-199a was localized in placental villi, mainly in the villus syncytiotrophoblast and villus matrix (**Figure 1A**). The expression of miR-199a was significantly up-regulated in the placenta of GDM patients of all ages (*P*<0.05, *P*<0.01, **Figure 1B**). The expression of miR-199a in the placenta detected by real-time fluorescence quantitative PCR was consistent with the results of *in situ* hybridization. The results showed that miR-199a was significantly upregulated in GDM patients of all ages compared with controls, especially in Y31-35 (*P*<0.01, **Figure 1C**). It is suggested that the expression level of miR-199a in the placenta may not be related to age, and the placental tissue may be involved in the occurrence of GDM.

### Overexpression of miR-199a Upregulates DNA Methylation Levels

**Figure 2:**
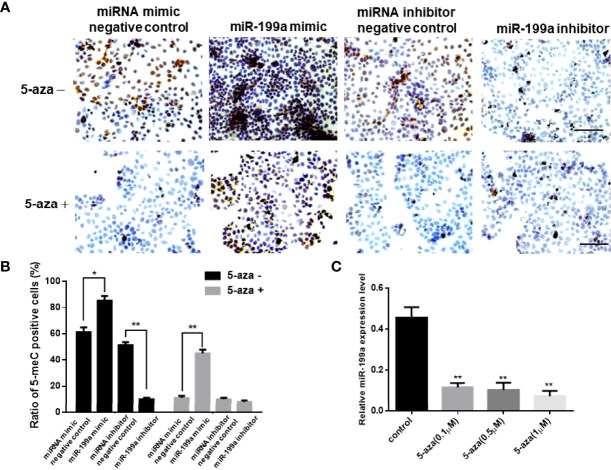
miR-199a mimic upregulates cellular methylation levels. **(A, B)** 5-aza immunohistochemical detection of the effects of miR-199a mimic and miR-199a inhibitor on the overall methylation level of JEG-3 cells. Brown is a positive signal. n=3. **(C)** Real-time PCR detection of miR-199a expression in JEG-3 cells treated with different concentrations of 5-aza. n=3. The scale bar indicates a distance of 100μm. *P<0.05; **P<0.01.

To investigate the role of miR-199a in GDM, we added miR-199a mimics to JEG-3 cells and performed immunohistochemical staining using the DNA methylation inhibitor 5-aza. The results showed that compared with the control, the proportion of 5-MeC positive cells in the cells transfected with miR-199a mimic was significantly increased (*P*<0.05). The proportion of 5-MeC-positive cells was significantly reduced in cells simultaneously transfected with miR-199a inhibitor (*P*<0.01, **Figures 2A, B**). Real-time PCR detected the expression of miR-199a, and the results showed that inhibiting DNA methylation significantly down-regulated the expression of miR-199a (*P*<0.01, **Figure 2C**). And the expression of miR-199a was hardly affected by the dose of 5-aza. This suggests that miR-199a regulates DNA methylation and may regulate the occurrence of GDM by regulating DNA methylation.

### MeCP2 Was a Target of miR-199a

**Figure 3:**
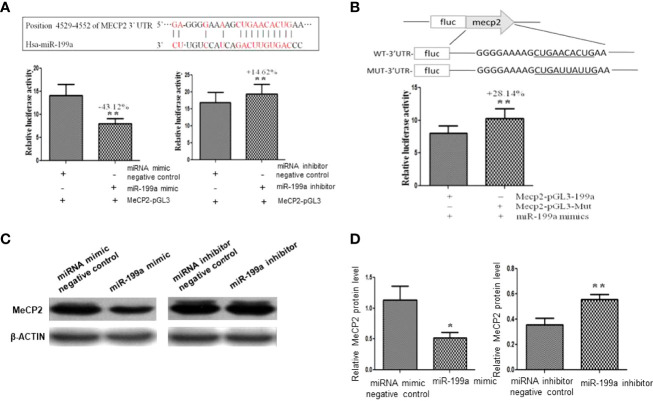
Target gene verification of miR-199a. **(A)** Prediction of the binding site of miR-199a and the 3’-UTR of MeCP2 mRNA and dual-luciferase detection of the target gene interaction relationship between miR-199a and the 3’-UTR of MeCP2 mRNA. n=3. **(B)** The inhibitory effect of miR-199a on MeCP2 was significantly reduced after induction of point mutations to the binding site. n=3. **(C, D)** Western-blot verification of the regulatory effect of miR-199a on the protein level of the target gene MeCP2. *P<0.05, **P<0.01.

To validate the targeted regulation of MeCP2 by miR-199a, we predicted the binding site of miR-199a in the 3’UTR of MeCP2 mRNA by PicTar and miRanda (**Figure 3A**). We cloned the wild-type-containing Mecp2 3’-UTR fragment downstream of the firefly luciferase reporter gene in the pGL3 control vector for dual-luciferase assay. The results showed that miR-199a mimics significantly reduced the expression of MeCP2, miR-199a inhibitor significantly increased the expression of MeCP2 (*P*<0.01, **Figure 3A**). To verify that miR-199a targets MeCP2, we constructed a base mutation vector targeting the site. The results of dual luciferase assay showed that the expression of MeCP2 in miR-199a mimic was significantly higher than that in the control group (*P*<0.01, **Figure 3B**). It is suggested that miR-199a regulates MeCP2 through the binding site. To verify the effect of miR-199a expression on the protein level of MeCP2, we performed Western blotting experiments. The results showed that miR-199a mimic inhibited the expression of MeCP2, and miR-199a inhibitor up-regulated the expression of MeCP2 (*P*<0.05, *P*<0.01, **Figures 3C, D**). The results show that miR-199a directly targets MeCP2 and regulates MeCP2 function.

### MeCP2 Was Downregulated in GDM Placenta

**Figure 4:**
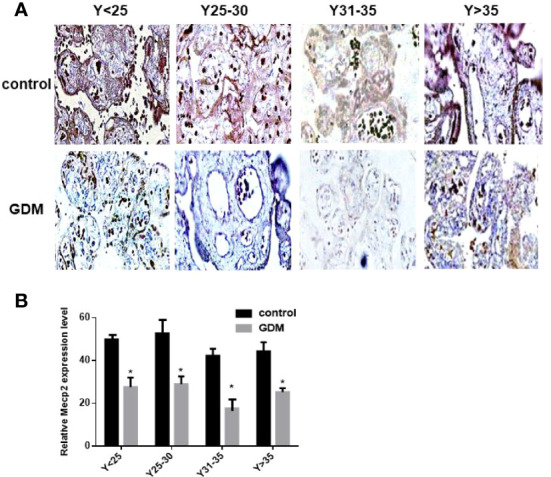
The expression of MeCP2 is down-regulated in the placenta of GDM patients. **(A, B)** Immunohistochemical assay to detect the expression of MeCP2 in the placenta of GDM patients. Brown is a positive signal. *P<0.05.

We detected the expression of MeCP2 in the placenta by immunohistochemical experiments, and the results showed that the expression of MeCP2 in the placenta of GDM patients was significantly down-regulated compared with the control group (*P*<0.05, **Figures 4A, B**). This corresponds to the up-regulated expression of miR-199a in the placenta of GDM patients detected by *in situ* hybridization experiments, suggesting that MeCP2 is a functional target gene of miR-199a.

### The Regulatory Effect of miR-199a on the MeCP2-TRPC3 Pathway

**Figure 5:**
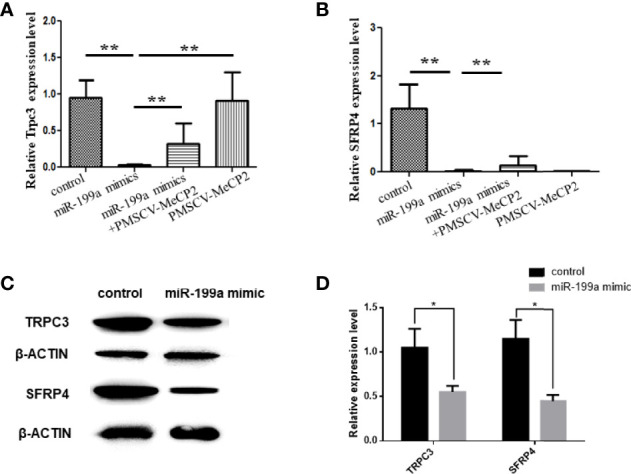
miR-199a regulates MeCP2-TRPC3 expression. **(A)** The expression of Trpc3 was detected by real-time PCR. n=10. **(B)** The expression of SFRP4 was detected by real-time PCR. n=10. **(C, D)** Western blot experiments to detect the expression levels of Trpc3 and SFRP4 in JEG3 cells transfected with miR-199a mimics. n=3, *P<0.05, **P<0.01.

To investigate the regulatory role of MeCP2 in GDM, we used Real-time PCR to detect the expression of TRPC3 and SFRP4 in JEG3 cells transfected with miR-199a and MeCP2. The results showed that compared with the control group, miR-199a mimic significantly inhibited the expression of TRPC3 and SFRP4 (*P*<0.01). After simultaneous transfection of miR-199a mimic and PMSCV-Mecp2 vectors, the expressions of TRPC3 and SFRP4 were significantly up-regulated (*P*<0.01, **Figures 5A, B**). It was suggested that the expression levels of Trpc3 and SFRP4 suppressed by miR-199a overexpression could be restored by Mecp2 dose compensation. Western blotting showed the same results that miR-199a mimic could inhibit the expression of TRPC3 and SFRP4 (*P*<0.05, **Figures 5C, D**). This suggests that miR-199a regulates the MeCP2-TRPC3 pathway by regulating MeCP2, thereby regulating the occurrence of GDM.

## Discussion

MeCP2, a key component of constitutive heterochromatin, functions as a repressor of gene transcription and reduces the expression of its target genes by binding to promoter regions and collecting histone deacetylase complexes (17). TRPC channels are a class of non-selective cation channels permeable to Ca^2+^ (18). Tang et al. found that TRPC3 could inhibit cardiac fibrosis in type 1 diabetes (19); Lang et al. found that TRPC3 could inhibit high glucose-induced endothelial cell proliferation and migration (20).; liu et al. found that TRPC could alleviate diabetic kidney injury (21). This suggests that the MeCP2-TRPC3 pathway is involved in the regulation of diabetes, so we verified the regulatory role of the differentially expressed miR-199a in GDM and MeCP2.

The results of *in situ* hybridization analysis showed that the expression of miR-199a-5p in the placental tissue of GDM patients was significantly higher than that of the control group, suggesting that the high expression of miR-199a-5p may be related to the occurrence of GDM. To further explore the possible mechanism of miR-199a-5p in GDM, we used bioinformatics methods to predict the target genes of miR-199a-5p, and found that MeCP2 has a response element recognized by miR-199a-5p, we used dual fluorescence The targeting regulation relationship between miR-199a-5p and MeCP2 was verified by the nephelase reporter system. The results of Real-time PCR and Western-blot methods showed that miR-199a-5p mimics down-regulated the expression of MeCP2, and miR-199a-5p inhibitor up-regulated the expression of MeCP2. MeCP2 is an activator of TRPC channel, and MeCP2 can specifically activate TRPC3. The TRPC3 pathway is known to be involved in regulating insulin-induced glucose uptake and utilization in muscle tissue through the regulation of Ca^2+^ (22, 23).

In order to further analyze the mechanism of miR-199a-5p and its target gene MeCP2 involved in the occurrence of GDM, we selected the downstream target gene TRPC3 of MeCP2 for detection. In the case of overexpression of miR-199a-5p, the expression of TRPC3 was down-regulated; in the case of overexpression of MeCP2, the expression of TRPC3 was up-regulated. It is suggested that miR-199a may participate in the occurrence of GDM by regulating the MeCP2-TRPC3 pathway.

## Data Availability Statement

The raw data supporting the conclusions of this article will be made available by the authors, without undue reservation.

## Ethics Statement

The full name of the Ethics Committee is “Ethics Committee of the National Family Planning Institute”, which is affiliated to the National Institute of Family Planning of China. The patients/participants provided their written informed consent to participate in this study.

## Authors Contributions

C-YG wrote the manuscript; J-LC, X-QZ and LZ performed molecular experiments. XM and H-FX provided funding and experimental guidance. All authors contributed to the article and approved the submitted version.

## Funding

This work was funded by grants from the National Natural Science Foundation of China (No. 32030103).

## Conflict of Interest

The authors declare that the research was conducted in the absence of any commercial or financial relationships that could be construed as a potential conflict of interest.

## Publisher’s Note

All claims expressed in this article are solely those of the authors and do not necessarily represent those of their affiliated organizations, or those of the publisher, the editors and the reviewers. Any product that may be evaluated in this article, or claim that may be made by its manufacturer, is not guaranteed or endorsed by the publisher.
